# Assessment of reliability and validity of the Spanish version of the Nursing Students' Perception of Instructor Caring (S-NSPIC)

**DOI:** 10.1371/journal.pone.0212803

**Published:** 2019-02-28

**Authors:** Macarena Romero-Martín, Juan Gómez-Salgado, Máximo de la Fuente-Ginés, Juana Macías-Seda, Alejandro García-Díaz, José Antonio Ponce-Blandón

**Affiliations:** 1 Centro Universitario de Enfermería Cruz Roja, University of Seville, Sevilla, Spain; 2 Department of Nursing, University of Huelva, Huelva, Spain; 3 Department of Nursing, University Espíritu Santo, Guayaquil, Ecuador; 4 Department of Nursing, University of Seville, Sevilla, Spain; Murray State University, UNITED STATES

## Abstract

The care that clinical instructors demonstrate to students is essential for their education, considering the strong impact it has on their future relationships with patients, relatives, and other health professionals. Nursing Students’ Perceptions of Instructor Caring (NSPIC) is an instrument designed to assess nursing students’ perceptions of instructor’s caring behaviors. A trans-cultural, conceptual, and psychometric validation study was conducted with 315 nursing students at the University of Seville during their clinical practices in three regional hospitals. The NSPIC was translated and adapted to Spanish. The content validity was established by a panel of experts. To assess concurrent validity the culturally adapted Spanish version of the Clinical Placement Evaluation Tool (CPET) was used as a gold standard. The construct validity was determined by an exploratory factor analysis to identify the internal structure of the NSPIC-S. The internal consistency was established by Cronbach’s α and the intra-observer reliability for each item was established by test-retest. The content validity index varied between 0.53 and 0.93 and the correlation to the CPET was moderate. The factor analysis revealed a structure of five factors, one of which differed from the original scale. The value of Cronbach’s α was 0.95 and intraclass correlation coefficients varied between 0.5 and 0.89. Our study provided a culturally adapted version of the NSPIC, valid and reliable to be used in the Spanish context, the NSPIC-S.

## Introduction

Caring is considered the core concept of nursing, the essence that guides nursing theory, practice and research [1]. Since caring is a fundamental value in nursing, the ability to care should be an aspiration for all nursing students [2]. Nursing curricula should be focused on caring and should offer students a general appreciation of caring so that they will be capable of performing their professional roles as caregivers [3]. However, given its subjective, ambiguous and complex nature, teaching the practice of caring is a difficult challenge. According to Watson, care occurs through an inter-subjective and transpersonal relationship, understood as a spiritual interconnection of mutual transformation between the caregiver and the person being cared for, which transcends both people to affect their realities and enrich their vision of the world [4]. Under this approach, and considering that the instructor-student relationship is based on caring, this relationship could be a valuable opportunity to learn how to care, since both incorporate new experiences and meanings to their spiritual dimension.

The clinical environment should provide appropriate interactions and feedback from the staff in order to improve students’ self-esteem and learning capabilities, help them integrate theoretical knowledge in clinical practice and their clinical judgment, and to encourage the development of their professional identity [5,6]. The caring relationship between the instructor and the student is the starting point and foundation for the learning process and for their developing as future professionals [7]. This relationship is based on respect [8,9], trust [10,11], mutual knowledge [10], and acceptance [8]. These values facilitate a safe [9,12], free [11], and supportive [7] relationship that encourages self-confidence [12] and empowerment [11] in the student and allows for a satisfying learning experience [8].

The role of the clinical instructor includes guiding, giving advice, teaching, and being a role model for their students [13]. The clinical instructor, as a role model, has the capacity to influence the student professionally and personally [14], as they imitate the attitudes and behaviors that students observe in their instructors [7,12].

The reciprocal relationship between students and instructors helps students grow as people who care for others, so nursing education is an opportunity to develop, improve, and promote caring behaviors in students [15,16]. Caring for the students teaches them how to care, so that in their interaction with the patient, they repeat the behavior of when they were being cared for by their instructors [11]. The care and respect that students receive from their instructors influence their self-image and drive them to become better caregivers. For this reason, educators should create a learning environment where students feel cared for, since this caring environment is what will mold them as future caregivers [6].

In short, the care that clinical instructors demonstrate to the students is essential for their education, given the strong impact it has on future relationships with patients, relatives, and other health professionals [17]. The assessment of caring behaviors that students perceive from their instructors allows for describing the type of care that they are internalizing.

The Nursing Students’ Perceptions of Instructor Caring (NSPIC) is an instrument designed to measure nursing student’s perceptions of their instructors’ caring behaviors. This perception was defined as “nursing students’ awareness of a mutual and reciprocal connection between the self and the instructor that enables them to reach for meaning and wholeness and grow as caring professional nurses” (Holland Wade and Kasper, 2006, p.164). The study involved a self-completed questionnaire of 31 items grouped into 5 domains: *confidence through caring*, *supportive learning climate*, *appreciation of life’s meaning*, *control versus flexibility*, *and respectful sharing*. Each item was evaluated using a Likert scale from 1 to 6. The scale demonstrated high reliability, with a Cronbach α of 0.97 [18] and has been trans-culturally adapted in China [19] and Italy [20].

### Objective

To evaluate the validity and reliability of the Spanish version of the NSPIC with nursing students in order to obtain an adequate instrument for assessing the care received from their clinical instructors.

## Methodology

### Study design and participants

A study of trans-cultural, conceptual and psychometric evaluation was conducted. It is recommended to have at least 10 subjects for each item, which in this case means having a minimum of 310 participants [21]. In order to be eligible for the study, students must have been enrolled at the University of Seville and must have completed at least one stage of clinical practice. These requirements were met by 360 possible student candidates for the study. The study sample was selected by convenience. It consisted of 315 nursing students from the University of Seville who attended class on the agreed days for the collection of data (participation rate 87.5%). The students carried out their clinical practices at one of the following hospitals: Virgen del Rocio, Virgen Macarena or Virgen de Valme; or at primary healthcare centers of the city of Seville (Spain). A total of 70 students were chosen from the study sample for the test-retest intra-observer reliability assessment. These 70 students were selected for having two compulsory lessons two weeks apart. This was highly convenient for the reliability test, as questionnaires were distributed twice to the same 70 students and their answers were compared. Two weeks is the recommended period of time between the two measures [22]. It should be clarified that attendance at the lessons chosen for the test-retest was mandatory, not so the participation in the study, which was completely voluntary.

### Translation process and cultural adaptation

The steps provided in the literature were followed: direct translation, synthesis, back translation, consolidation, and pre-test [22]. Initially, two bilingual translators whose native language was Spanish, a nurse and English philologist, completed a first translation of the NSPIC into Spanish independently. The research team unified the two versions of the translators and received the translators’ approval for a first Spanish version of the NSPIC. A back translation into English was completed by two different bilingual translators whose native language was English, with the same background as the previous two, a nurse and a philologist. The back translators worked independently and without the original version of the scale. Both translations were performed in search of conceptual semantic equivalence and paying close attention to both nursing technicalities (spiritual dimension of life, caring needs) and colloquial language (caught up). A multidisciplinary committee was formed by a bilingual expert in methodology, a bilingual expert in nursing theoretical framework, the four translators, and the research team (the authors of the present article: four PhD nurses, one clinical nurse and one lecturer academic nurse). The objective of this committee was to compare the four translations, the unified version obtained by direct translation and the original version, coming to an agreement by consensus regarding ambiguities and discrepancies. The first prototype of NSPIC-S was distributed among a panel of 15 experts who knew in depth the concept of research at an academic and/or professional level, met the characteristics of the users of the scale (nursing students), and had at least 5 years of experience [21]. The panel of experts assessed the clarity of the NSPIC-S prototype and made open comments with suggestions for improvement.

### Validity

A valid scale measures the concept for which it has been designed. Content validity refers to the extent to which a measure represents all facets of a given construct, criterion validity refers to the extent to which a measure is related to an outcome, and construct validity refers to the degree to which the results can be related to a measurement of the studied phenomenon [22]. The content validity of the NSPIC-S was assessed by a panel of experts formed by 5 clinical nurses, 5 academic nurses and 5 nursing students.The experts panel evaluated the relevancy and suitability of each survey item on a 4-point Likert scale where 1 meant not relevant, 2 somewhat relevant, 3 moderately relevant, and 4 very relevant. For the criterion validity, a culturally adapted Spanish version of the Clinical Placement Evaluation Tool (CPET) was used as a *gold standard*. The CPET is a scale designed to measure students´ evaluation of their clinical practices. It consists of 17 items that represent three dimensions: *characteristics of the instructor and the process of instructing*, *team integration*, *and transmission of information*. Each item is evaluated on a 5-point Likert scale, ranging from 17–85 as total score, in which the lowest score means a more positive perception of the place of clinical practices. The construct validity was determined using exploratory factor analysis to identify the internal structure of the NSPIC-S.

### Reliability

Internal consistency was established calculating Cronbach´s α for the scale and all its dimensions, and confirmed by the split-half coefficient, for which the items were distributed in two equal halves with the same number of items for each dimension. The intra-observer reliability for each item was established by test-retest.

### Procedure

The NSPIC-S and the Spanish version of the CPET were handed out to participants in person. A researcher was present during the collection of data to answer any questions and to ensure rigor and meticulousness in the completion of the questionnaires. The participants were given an additional questionnaire to obtain socio-demographic characteristics. A group of 70 students filled out the NSPIC-S again after two weeks for the test-retest. The answers were recorded anonymously in a database.

### Statistical analysis

Descriptive statistics were used to summarize socio-demographic variables. Regarding the expert panel, the Content Validity Index for each item (CVI-I) and the total of the scale (CVI-S) was calculated. The CVI-I was computed as the number of experts who considered the item relevant, this is to say, who rated it as 3 or 4 on the Likert scale, divided by the total number of experts. The CVI-S was calculated as the average of CVI-Is. A CVI-I above 0.78 and a CVI-S above or equal to 0.9 were considered acceptable [21]. Normality distribution was assessed using the Kolmogorov-Smirnov test, resulting in a value of p<0.001 for the scores of the NSPIC-S and the CPET. In relation to criterion validity, the Spearman correlation coefficient was used for correlating the scores of the NSPIC-S and its subscales and the scores of the CPET gold standard questionnaire and its subscale *characteristics of the instructor*. For the construct validity, an Exploratory Factor Analysis (EFA) was used, a previous verification of the suitability was checked with the Bartelett sphericity test and the Kaiser-Meyer-Olkin (KMO) coefficient. A significance of p<0.05 for the Bartelett test and a value of KMO>0.60 were considered acceptable as recommended in the literature [23]. In the EFA the method of maximum probability for the extraction of data and the Varimax rotation were used. It was considered a significant association when the values of the EFA were above 0.30 [23]. In relation to reliability, the item-total correlation index was calculated, with values above 0.2 considered acceptable [24]. Cronbach´s α was used to determine internal consistency with values above 0.7 considered acceptable [25]. The Guttman Split-half coefficient was also considered for reliability confirmation [23]. With the results of the test-retest, the Intraclass Correlation Coefficient (ICC) was calculated, considering poor correlation values below 0.40 [23]. In all calculations, the computer program SPSS version 16.0 was used.

### Ethical considerations

Participants were previously informed about the purpose of the study and they provided informed voluntary written consent. Their information was registered anonymously so it would not be possible to identify participants´ answers. The study was given consent from the directors of the Schools of Nursing, Physical Therapy, and Podiatry of the University of Seville. Authorization was obtained from the authors of the original NSPIC scale and the Spanish version of the CPET for its adaptation and use in this present study. Modification suggested for the S-NSPIC were approved by the authors of the original scale. This research was approved by the Ethic Committee of Research at the Red Cross Nursing School, University of Seville.

## Results

Of the 315 surveys collected, 310 were considered valid, and 4 were excluded, since they referred to practices during international exchange, and one for being completed incorrectly. Nursing students who made their clinical practice abroad were excluded because we aimed to validate the NSPIC within the Spanish culture, and their answers would refer to a different context. The final response rate was 98.4%. The socio-demographic characteristics of the participants are shown in Table 1. Sample was predominantly female, mean age 22.38, Standard Deviation 3.53.

**Table 1:** Sample characteristics (N = 310).

Variables		n	%
Gender			
	Female	270	87.9
	Male	37	12.1
Level of studies			
	Third year	121	39.0
	Fourth year	189	61.0
Marital status	Single	300	98.0
	Married	6	2.0
Clinical placement			
	Virgen Macarena Hospital	104	34.1
	Virgen Rocío Hospital	58	19.0
	Virgen Valme Hospital	29	9.5
	Primary Health Center	114	37.4
Previous experience in caring			
	No	257	83.4
	Yes	51	16.6

### Validity

With respect to content validity, all CVI-I values were > 0.78 except for item 12 and item 16 (Table 2).

**Table 2:** Content validity index (CVI-I), intraclass correlation coefficient (ICC) and item-total correlation for NSPIC-S (n = 310).

Item	CVI-I	ICC	Item-total correlation
1. Shows genuine interest in patients and their care.	0.93	0.83	0.63
2. Displays kindness to me and others.	0.93	0.80	0.72
3. Instills in me a sense of hopefulness for the future.	0.87	0.83	0.68
4. Makes me feel that I can be successful.	0.87	0.79	0.69
5. Helps me envision myself as a professional nurse.	0.93	0.82	0.70
6. Makes me feel like a failure.	0.80	0.84	0.30
7. Does not believe in me.	0.80	0.50	0.49
8. Cares about me as a person.	0.87	0.81	0.71
9. Respects me as an unique individual.	0.87	0.78	0.58
10. Is attentive to me when we communicate.	0.87	0.84	0.71
11. Inappropriately discloses personal information about me to others.	0.93	0.72	0.37
12. Does not reveal any of his or her personal side.	0.67	0.54	0.35
13. Acknowledges his or her own limitations or mistakes.	0.87	0.60	0.53
14. Makes himself or herself available to me.	0.93	0.74	0.67
15. Clearly communicates his or her expectations.	0.93	0.68	0.59
16. Serves as a trusted resource for personal problem solving.	0.53	0.78	0.69
17. Offers support during stressful times.	0.93	0.85	0.74
18. Accepts my negative feelings, while helping me to see the positive.	0.87	0.81	0.75
19. Allows me to express my true feelings.	0.87	0.83	0.76
20. Discourages independent problem solving.	0.93	0.75	0.68
21. Inspires me to continue my knowledge and skill development.	0.93	0.89	0.75
22. Makes me nervous in the clinical laboratory.	0.93	0.81	0.74
23. Does not trust my judgment in the clinical laboratory.	0.87	0.66	0.46
24. Seems caught up in his or her own priorities, rather than responding to my needs.	0.87	0.76	0.69
25. Makes demands on my time that interfere with my basic personal needs.	0.80	0.65	0.46
26. Focuses on completion of patient care tasks, rather than the patient’s needs.	0.87	0.72	0.53
27. Helps me find personal meaning in my experiences.	0.80	0.84	0.69
28. Encourages me to see others’ perspectives about life.	0.80	0.80	0.69
29. Helps me understand the spiritual dimensions of life.	0.87	0.68	0.54
30. Is inflexible when faced with unexpected situations (happenings).	0.80	0.50	0.32
31. Uses grades to maintain control of students.	0.80	0.81	0.11

In relation to criterion validity, the absolute values of the Spearman correlation coefficient varied between 0.536 and 0.725 (Table 3).

**Table 3:** Spearman correlation coefficients between the scores of the NSPIC-S and its subscales and between the CPET gold standard questionnaire and its subscale characteristics of the preceptor.

	Total CPET	Characteristics of the preceptor (CPET subscale)
Total NSPIC-S	-0.725*	-0.715*
Confidence trhough caring	-0.629*	-0.633*
Supportive learning climate	-0.635*	-0.616*
Appreciation of life's meanings	-0.586*	-0.559*
Control versus flexibility	0.536*	0.547*
Professional anticipation	-0.666*	-0.670*

* p<0,001

Note: Negative values are interpreted as a positive correlation since the scales are graduated inversely.

Before the EFA, KMO = 0.944 and p<0,001 was achieved in the Bartelett test. The EFA results revealed five factors which accounted for 60.66% of the total variance, and a factor loading above 0.3 in all the items except for items 12 and 31, whose factor loading was 0.29 and 0.26, respectively(Table 4).

**Table 4:** Factor analysis and internal consistency results for NSPIC-S (component loading and Cronbach's alpha coefficient N = 310.

	Factor 1	Factor 2	Factor 3	Factor 4	Factor 5
**Confidence through caring** (Cronbach's α = 0.896)					
9. Respects me as an unique individual.	**0.532**	0.029	0.365	0.160	-0.219
13. Acknowledges his or her own limitations or mistakes.	**0.529**	0.243	0.201	0.083	-0.099
2. Displays kindness to me and others.	**0.521**	0.105	0.430	0.353	-0.201
1. Shows genuine interest in patients and their care.	**0.519**	0.279	0.209	0.288	-0.110
21. Inspires me to continue my knowledge and skill development.	**0.506**	0.384	0.248	0.428	-0.152
14. Makes himself or herself available to me.	**0.504**	0.166	0.423	0.165	-0.268
10. Is attentive to me when we communicate.	**0.482**	0.109	0.456	0.273	-0.295
11. Inappropriately discloses personal information about me to others.	**-0.473**	-0.044	-0.015	-0.079	0.205
7. Does not believe in me.	**-0.472**	-0.049	-0.118	-0.179	0.295
22. Makes me nervous in the clinical laboratory.	**0.466**	0.235	0.415	0.352	-0.210
20. Discourages independent problem solving.	**0.458**	0.362	0.285	0.343	-0.078
30. Is inflexible when faced with unexpected situations (happenings).	**-0.353**	-0.100	-0.042	-0.054	0.132
6. Makes me feel like a failure.	**-0.347**	0.005	-0.061	-0.077	0.216
**Appreciation of life's meanings** (Cronbach's α = 0.888)					
27. Helps me find personal meaning in my experiences.	0.185	**0.781**	0.248	0.255	-0.127
28. Encourages me to see others’ perspectives about life.	0.205	**0.779**	0.250	0.246	-0.106
29. Helps me understand the spiritual dimensions of life.	0.066	**0.749**	0.232	0.177	-0.034
**Supportive learning climate** (Cronbach's α = 0.903)					
19. Allows me to express my true feelings.	0.242	0.399	**0.652**	0.288	-0.142
16. Serves as a trusted resource for personal problem solving.	0.036	0.441	**0.645**	0.249	-0.232
18. Accepts my negative feelings, while helping me to see the positive.	0.289	0.406	**0.618**	0.286	-0.098
17. Offers support during stressful times.	0.437	0.351	**0.550**	0.157	-0.136
8. Cares about me as a person.	0.254	0.346	**0.521**	0.308	-0.195
**Professional anticipation** (Cronbach's α = 0.852)					
4. Makes me feel that I can be successful.	0.242	0.239	0.240	**0.775**	-0.142
3. Instills in me a sense of hopefulness for the future.	0.205	0.348	0.209	**0.689**	-0.187
5. Helps me envision myself as a professional nurse.	0.298	0.251	0.335	**0.658**	-0.091
15. Clearly communicates his or her expectations.	0.267	0.306	0.303	**0.315**	-0.166
**Control versus flexibility** (Cronbach's α = 0.741)					
25. Makes demands on my time that interfere with my basic personal needs.	-0.242	-0.065	-0.086	-0.099	**0.678**
24. Seems caught up in his or her own priorities, rather than responding to my needs.	-0.332	-0.243	-0.267	-0.214	**0.590**
26. Focuses on completion of patient care tasks, rather than the patient’s needs.	-0.249	-0.327	-0.096	-0.040	**0.580**
23. Does not trust my judgment in the clinical laboratory.	-0.214	-0.167	-0.186	-0.131	**0.389**
12. Does not reveal any of his or her personal side.	-0.026	-0.084	-0.214	-0.209	0.294
31. Uses grades to maintain control of students.	-0.132	0.103	-0.010	0.007	0.260
**Eigenvalue**					
% of varibility	41.719	7.216	4.355	3.746	3.630
% of cumulative variability	41.719	48.935	53.289	57.036	60.665

In bold = factor loading above 0.3.

The dimension *respectful sharing* was not confirmed, as items 9 and 10 were related to the dimension *confidence through caring*, and item 12 had a factor loading less than 0.3. Compared with the original structure the EFA showed a new factor comprised of items 3, 4, 5 and 15. Some changes from the original tool were identified regarding the components of the dimensions, such as item 8 that is now related to *supportive learning climate*, items 23 and 24 which are now loaded in *control vs flexibility*, items 11, 13, 14, 20 and 30 are now included in *confidence through caring*, and item 31 which had a factor loading lower than 0.3.

### Reliability

Cronbach´s alpha coefficient of the NSPIC-S was 0.951 (Table 4). The Guttman Split-half coefficient was 0.862. The ICC varied between 0.5 and 0.89 and the item-total correlation coefficient values were all higher than 0.2, except for item 31 (Table 2).

## Discussion

The results revealed a culturally adapted version of the NSPIC, valid and reliable to be used in the Spanish context, the NSPIC-S.With respect to content validity, items 12 (i.e., *does not reveal any of his or her personal side*) and 16 (i.e., *serves as a trusted resource for personal problem solving*) had an insufficient CVI-I, which represents a disagreement in the relevancy of the item among the panel of experts. Both items refer to an approach at a personal level between the instructor and the student. This discrepancy could be explained if instructor-student relationship is interpreted as learning and growing processes that develop only at a professional level for both. In the power relationship between instructor and student, instructors are aware of their position of superiority [10] which can place a barrier that impedes theinstructor-student relationship from advancing in the personal realm. Moreover, the demonstration of procedural-type practical abilities is valued by both, instructors and students [26], which infers instructor-student relationship with more of a professional nature rather than personal. Some authors suggest previous education for students about how to establish professional relationships with instructors to improve the quality of their learning experience [27].

The internal structure of the scale consisted of 5 factors, although there were conceptual differences in the dimensions from the original NSPIC. The dimension *respectful sharing* cannot be considered in the Spanish version since its items are included in dimension *confidence through caring*. Mutual respect is considered a previous requirement in the instructor-student relationship [17]. An environment of respect and trust is the appropriate context in which to start a relationship where the student feels safe, protected, and motivated to learn [9]. Respect is a key element for building an instructor-student relationship and it is part of the expectations of both people [8]. Therefore, because respect is an essential component in the instructor-student relationship, it may be possible that in the present study, *respectful sharing* was not considered a factor of care transmitted by the instructors, but as an intrinsic characteristic tied to the very existence of the relationship.

On the other hand, *confidence through caring* was the most valued dimension in previous studies carried out with the NSPIC [28–32]. Labrague et al. (2015) identified the association between the caring behavior of instructors and students and described the instructos's impact on the students. The results revealed a strong correlation between the dimensions *confidence through caring* and the students’ caring behaviors [33]. According to Gibbs and Kuling (2017), instructors can promote students´ self-confidence in creating a safe atmosphere, sharing knowledge, and providing feedback. This self-confidence makes the students feel more competent, they participate in a more meaningful way, and they improve in their practices. Furthermore, Heydari et al. (2016) showed that self-confidence in students increased their capacity to develop effective relationships and nursing care in the clinical environment. Álvarez and Moya (2017) noted that self confidence in students instilled by their clinical instructors generated responsibility, freedom, independence, and helped them develop clinical reasoning, make decisions, and progress towards an autonomous practice. All that is mentioned above reveals the significant relevance of the dimension *confidence through caring* in the instructor-student relationship. This relevance might explain that in the NSPIC-S the dimension *respectful sharing* resulting in this present study, as well as items *inappropriately discloses personal information about me to others*, *acknowledges own limitations or mistakes*, *makes herself/himself available to me*, *discourages independent problem solving and is inflexible when faced with unexpected situations; resulted included in* the dimension *confidence through caring*.

The results of this present study revealed an internal structure that includes a new factor made up of the items *instills in me a sense of hopefulness for the future*, *makes me feel that I can be successful*, *helps me to envision myself as a professional nurse and clearly communicates her/his expectations*. This new dimension could be called *professional nurse autonomy* as it refers to the visualization of the student as a nurse and brings awareness to the student´s professional potential in independent decision-making roles in defending patient care [34]. These items motivate students to do introspective and visualization exercises that allow them to have faith in themselves as future professionals. The dimension *professional nurse autonomy refers to* getting to know oneself in the professional sense and recognizing positive feelings when having attained an achievement and negative feelings in the face of difficulties, which the instructor helps them to exteriorize and accept. Therefore, the new dimension is related to three of the *Carative Factors* of Jean Watson: *enabling and sustaining faith and hope*, *sensitivity to self and others* and *promoting and accepting the expression of positive and negative feelings and emotions* [35]. Positive experiences during clinical practice are an essential element in acculturation into the nursing profession [5]. The dimension *professional nurse autonomy* is supported by studies that show that clinical instructors have a strong influence in the professional socialization of nursing students. Teaching styles and interaction with instructors influences the way in which students immerse themselves socially within the profession [5,36,37].

In addition to modifications in the dimensions of the scale, our results also suggested modification in certain items. Item *cares about me as a person* of dimension *confidence through caring* was part of dimension *supportive learning climate*. This result coincided with the study of trans-cultural adaptation of the NSPIC to Italian [20]. The authors attributed the new location of the item to a lack of differentiation among the two dimensions by students. Support is a basic element of care. The revision carried out by Drahošová and Jarošová (2016) about qualitative studies of nursing care concluded that caring is a specific interpersonal process whose result is protection, emotional support, and attention to the needs of the other person [38]. It is therefore understandable that students’ perception of feeling cared for as a person (item 8) was associated with the dimension that refers to a supportive environment.

The items *does not trust my judgement in the clinical laboratory* and *seems caught up in her/his own priorities rather than responding to my needs* were included in dimension *control vs flexibility* in the NSPIC-S. This result coincides with the study of the trans-cultural adaptation of the NSPIC to Chinese [19]. The educational relationship between instructor and student demands a context of mutual trust [9]. This is manifested in what Rivera Álvarez and Medina Moya (2017) called the *virtuous cycle of trust*: the nurse shows trust in the student, and the student imitates and reciprocates this behavior while building self-confidence at the same time, which leads the way to autonomous practice. In other words, the lack of trust in the student can interfere in the development of his/her independence and will require more control of the student’s progression from the instructor. This trusting approach could explain the placement of item *does not trust my judgement in the clinical laboratory* in the S-NSPIC.

Item 12 (i.e., *does not reveal any of his or her personal side*) and item 31 (i.e., *uses grades to maintain control of students*) was not associated significantly with any of the dimensions of the scale. In addition, item 12 had an insufficient CVI-I which lacks agreement among the judges of the panel with regard to its relevancy. Item 31 had an item-total correlation coefficient below the acceptable value, which means it is not correlated with the rest of the items of the scale. In the internal organization of the clinical practices in which this study was conducted, it is common for an instructor not to establish final grades for students. Consequently, these grades can not be used as a tool to control, this explains why item 31 result isolated. Upon evaluating the results and considering that they are not acceptable according to the limits stablished by the researchers in more than one parameter for items 12 and 31, the research team decided by consensus to omit both items in the final version of the NSPIC-S.

## Conclusions

We pursued the trans-cultural adaptation of the NSPIC to the Spanish context. In light of the results, we concluded that the NSPIC-S had satisfactory values of validity and reliability and it was a suitable instrument for the evaluation of students´ perception of instructor caring behaviors. The resulting version of the scale consists of 29 items grouped into 5 factors: *confidence through* caring (13 items), *supportive learning climate* (5 items), *appreciation of life meaning* (3 items), *control versus flexibility* (4 items) and *professional nurse autonomy* (4 items). The use of the NSPIC-S in studies that evaluate education in a clinical environment could help in grasping the model of care that students are receiving and the identity of the role they are interiorizing. The results of the studies using the NSPIC-S can help improve educational work of clinical instructors, giving them a deeper understanding and raising their awareness about the impact and scope of the relationship they establish with students and promoting caring behaviors that serve as a learning model. In addition, using the NSPIC-S in future research will allow us to design strategies to improve practices that are in line with the students´ perception that contribute to improve learning outcomes.

## Limitations

Firstly, the convenience sampling method limited generalization of the results to the Spanish nursing students population. All participants were students at the same university and therefore, may not be representative of the Spanish population of nursing students. Secondly, exploratory factor analysis was used to identify associations between variables when studied in the Spanish cultural context, without considering the original NSPIC structure as a starting point. Because the results describe a structure different from the original, performance of a confirmatory factor analysis is needed to substantiate the structure of the NSPIC-S that we obtained. We recommend further study with a larger population that includes Spanish students from multiple universities to conduct a confirmatory factor analysis. The resulting internal structure would be more reliable and valid for the Spanish context. Thirdly, our study sample was not homogeneous with regard to gender, with a majority of female students and a majority of female staff at the hospitals where the practices were carried out. Performing multicenter randomized studies with a homogeneous sample of participants is recommended.

## Supporting information

S1 Text NSPIC original version in English. (DOCX)

S2 Text NSPIC-S transcultural adapted version in Spanish. (DOCX)
