# Supplementary material for: Assessment of reliability and validity of the Spanish version of the Nursing Students' Perception of Instructor Caring (S-NSPIC)
Source: PLoS One. 2019 Feb 28;14(2):e0212803. doi: 10.1371/journal.pone.0212803 (PMC6394939; doi:10.1371/journal.pone.0212803)
Supplement: S1 Text — (DOCX) [file pone.0212803.s001.docx]

**NURSING STUDENTS’ PERCEPTIONS OF INSTRUCTOR CARING**

My instructor:

1. Shows genuine interest in patients and their care.
2. Displays kindness to me and others.
3. Instills in me a sense of hopefulness for the future.
4. Makes me feel that I can be successful.
5. Helps me envision myself as a professional nurse.
6. Makes me feel like a failure.
7. Does not believe in me.
8. Cares about me as a person.
9. Respects me as an unique individual.
10. Is attentive to me when we communicate.
11. Inappropriately discloses personal information about me to others.
12. Does not reveal any of his or her personal side.
13. Acknowledges his or her own limitations or mistakes.
14. Makes himself or herself available to me.
15. Clearly communicates his or her expectations.
16. Serves as a trusted resource for personal problem solving.
17. Offers support during stressful times.
18. Accepts my negative feelings, while helping me to see the positive.
19. Allows me to express my true feelings.
20. Discourages independent problem solving.
21. Inspires me to continue my knowledge and skill development.
22. Makes me nervous in the clinical laboratory.
23. Does not trust my judgment in the clinical laboratory.
24. Seems caught up in his or her own priorities, rather than responding to my needs.
25. Makes demands on my time that interfere with my basic personal needs.
26. Focuses on completion of patient care tasks, rather than the patient’s needs.
27. Helps me ﬁnd personal meaning in my experiences.
28. Encourages me to see others’ perspectives about life.
29. Helps me understand the spiritual dimensions of life.
30. Is inﬂexible when faced with unexpected situations (happenings).
31. Uses grades to maintain control of students.
