# Supplementary material for: Assessment of reliability and validity of the Spanish version of the Nursing Students' Perception of Instructor Caring (S-NSPIC)
Source: PLoS One. 2019 Feb 28;14(2):e0212803. doi: 10.1371/journal.pone.0212803 (PMC6394939; doi:10.1371/journal.pone.0212803)
Supplement: S2 Text — (DOCX) [file pone.0212803.s002.docx]

**SPANISH NURSING STUDENTS’ PERCEPTIONS OF INSTRUCTOR CARING**

Mi instructor:

1. Muestra verdadero interés por los/las pacientes y sus cuidados.
2. Muestra amabilidad hacia mí y hacia los/as demás.
3. Me infunde un sentimiento de esperanza hacia mi futuro laboral.
4. Me hace sentir que puedo tener éxito en mi carrera profesional.
5. Me ayuda a visualizarme como enfermera/o profesional.
6. Me hace sentir que no soy apto/ a para la enfermería.
7. No cree en mí como profesional de enfermería.
8. Se preocupa por mí como persona.
9. Me respeta como persona.
10. Es atento/a cuando se comunica conmigo.
11. Revela mi información personal a los/as demás de manera inapropiada.
12. Reconoce sus propias limitaciones y/o errores.
13. Está a mi disposición cuando es necesario.
14. Comunica de forma clara sus expectativas respecto a los resultados de aprendizaje (qué debo aprender exactamente, cuál es mi rol como estudiante…).
15. Es de utilidad para resolver problemas personales.
16. Ofrece su apoyo en situaciones de tensión.
17. Acepta mis sentimientos negativos al mismo tiempo que me ayuda a ver lo positivo.
18. Me permite expresar mis verdaderos sentimientos.
19. Alienta la resolución autónoma de problemas.
20. Me inspira para continuar con el desarrollo de mis conocimientos y habilidades.
21. Me transmite tranquilidad en el ambiente clínico (prácticas clínicas, talleres de procedimientos, simulación…).
22. No confía en mi criterio en el ambiente clínico (prácticas clínicas, talleres de procedimientos, simulación…).
23. Parece centrarse más en sus prioridades que en responder a mis necesidades.
24. Me pide que lleve a cabo actuaciones que interfieren con mis necesidades personales (ejecutar tareas no propias de la profesión enfermera…).
25. Se centra en completar las tareas de cuidado más que en las necesidades propias de los pacientes.
26. Me ayuda a buscar el significado personal a mis experiencias.
27. Me alienta a ver las perspectivas de otras personas sobre la vida.
28. Me ayuda a entender la dimensión espiritual de la vida.
29. Es inflexible cuando se enfrenta a situaciones (sucesos) inesperados.
